# Rising Prevalence of Multidrug-Resistant *Klebsiella* spp. in Urinary Tract Infections: A Study from Doboj Hospital

**DOI:** 10.3390/antibiotics14121234

**Published:** 2025-12-07

**Authors:** Dragana Drakul, Bojan Joksimović, Ana V. Pejčić, Radica Živković-Zarić, Siniša Marić, Biljana Mijović, Tanja Ivanović, Dragana Erbez, Dragana Sokolović

**Affiliations:** 1 Department of Pharmacology, Faculty of Medicine Foča, University of East Sarajevo, 73300 Foča, Bosnia and Herzegovina; 2 Centre for Biomedical Research, Faculty of Medicine Foča, University of East Sarajevo, 73300 Foča, Bosnia and Herzegovina; 3 Department of Preclinical Subjects (Pathophisiology), Faculty of Medicine Foča, University of East Sarajevo, 73300 Foča, Bosnia and Herzegovina; 4 Department of Pharmacology and Toxicology, Faculty of Medical Sciences, University of Kragujevac, 34000 Kragujevac, Serbia; 5 Bolnica “Sveti Apostol Luka”, 74000 Doboj, Bosnia and Herzegovina; 6 Dapartment of Epidemiology, Faculty of Medicine Foča, University of East Sarajevo, 73300 Foča, Bosnia and Herzegovina; 7 Department of Pediatric and Preventive Dentistry, Faculty of Medicine Foča, University of East Sarajevo, 73300 Foča, Bosnia and Herzegovina

**Keywords:** multidrug resistance, *Klebsiella* spp., urinary tract infections, antibiotic, antimicrobial resistance

## Abstract

Urinary tract infections, as one of the most common infectious diseases, contribute substantially to the global healthcare burden, particularly due to the rising prevalence of resistant bacterial strains such as *Klebsiella pneumoniae*. **Background/Objectives**: The aim was to investigate the prevalence of urinary tract infection pathogens among hospitalized patients at Saint Apostol Luka Hospital in Doboj during the period 2021–2023. **Methods**: This retrospective cross-sectional study was conducted at Saint Apostol Luka Hospital, Doboj, Republic of Srpska, Bosnia and Herzegovina. Data from the Department of Microbiology were analyzed for the period 2021–2023, including patients with positive urine cultures (≥10^3^ CFU/mL) of a single uropathogen. Bacterial identification and susceptibility testing were performed according to EUCAST standards, and statistical analysis was carried out using SPSS v24. **Results**: *Escherichia coli* was the most frequent isolate (29.2%), followed by *Klebsiella* spp. (24.2%) and *Enterococcus* spp. (19.8%). A significant rise in *K. pneumoniae* prevalence and resistance to multiple antibiotics—including β-lactams, carbapenems, aminoglycosides, and colistin—was observed during the study period. **Conclusions**: This study revealed that *E. coli* and *Klebsiella* spp. were the leading uropathogens, with notable differences in distribution by sex, age, and hospital department. A marked rise in multidrug resistance, particularly among *K. pneumoniae*, was observed across the study period. These findings underscore the urgent need for continuous surveillance and stronger antimicrobial stewardship to curb resistance trends.

## 1. Introduction

Infectious diseases have always been considered a serious health threat. With the discovery of antibiotics, mortality caused by infectious diseases has significantly decreased. However, these diseases are re-emerging due to the uncontrolled use of antibiotics and the development of resistance to them. Due to the increasing rate of antibiotic resistance, urgent action is required. Urinary tract infections (UTIs) are the second most common infectious diseases after upper respiratory tract infections [1]. They are more common in women than in men, both in non-recurrent and recurrent cases. More than 50% of all women—and at least 12% of men—will experience an UTIs during their lifetime [2]. Uncomplicated UTIs are more common in sexually active younger women with anatomically and physiologically normal urinary tracts, whereas complicated infections occur more frequently in individuals who have another underlying condition (such as prolonged antibiotic use or diabetes), structural abnormalities of the urinary tract, the presence of a foreign body (like a catheter or calculus), and so on [3].

Gram-negative bacteria are responsible for approximately 90% of UTIs, while Gram-positive bacteria account for only about 10% of cases [3]. Among the pathogens causing UTIs, *Escherichia coli* is the most prevalent, accounting for approximately 75–90% of uncomplicated, community-acquired cases [4,5]. In contrast, bacteria such as *Pseudomonas* spp. and *Klebsiella* spp. are more commonly associated with complicated UTIs [6]. In hospital-acquired infections, *E. coli* is still significant, responsible for an estimated 30–50% of cases [7].

*Staphylococcus saprophyticus* is responsible for approximately 5–10% of UTIs in community-acquired, uncomplicated cases. By contrast, other bacterial causes such as *Klebsiella* spp., *Proteus* spp., *Pseudomonas* spp., and *Enterobacter* spp. are typically associated with urinary tract abnormalities or the presence of urinary catheters—often seen in complicated infections [8,9].

*Klebsiella pneumoniae* is one of the most frequent microorganisms causing UTIs in clinical settings [10]. Over the past two decades, its clinical importance has grown—driven by rising resistance rates and an increase in severe complications linked to this pathogen [11,12]. *K. pneumoniae* ability to form biofilms on medical devices—particularly urinary catheters—is a critical step in the pathogenesis of disease. Biofilm formation provides a protected environment that significantly enhances *K. pneumoniae* intrinsic resistance to antibiotics and shields it from host immune defenses, making infections especially persistent and difficult to eradicate, particularly in catheter-associated UTIs. The rapid rise in resistance among Klebsiella isolates may reflect the spread of ESBL-producing strains (particularly CTX-M types) and carbapenemase-producing lineages (KPC, NDM), which are increasingly documented across Eastern and Southeastern Europe. Porin mutations and upregulated efflux pumps further contribute to reduced susceptibility to β-lactams and carbapenems [13].

Moreover, global trends in multidrug resistance (MDR) and pandrug resistance (PDR) among *K. pneumoniae* complicate clinicians’ efforts to deliver rapid and effective therapy, contributing substantially to increased morbidity and mortality in patients [14].

A lack of proper diagnosis and timely treatment can lead to severe complications, such as disorders of the urinary tract, renal parenchymal damage, and uremia. In pregnant women, UTIs can result in preterm birth or even miscarriage. According to statistics from international organizations, 17–29 billion USD is spent annually on treating hospital-acquired infections worldwide—with approximately 39% attributable to UTIs [3].

Antibiotic treatment is the main cornerstone of any bacterial infection, including UTIs. However, in recent decades, the irresponsible use of antibiotics not only in human medicine, but also in veterinary medicine and the agro-industry, has led to a rise in bacterial resistance to antibiotics [15,16]. An unsuccessfully treated UTIs can lead to recurrent infections significantly impairing the patient’s quality of life or may become complicated by urosepsis [17]. For appropriate and effective empirical therapy, it is essential to be aware of the local resistance rates. Furthermore, studying local resistance is essential for identifying proper measures to halt the growth of antimicrobial resistance.

Despite extensive global surveillance, there remains substantial heterogeneity in UTIs pathogen distribution and antimicrobial resistance patterns at local and regional levels. Hospital-level data are essential because antimicrobial resistance is strongly influenced by institution-specific antibiotic prescribing, patient comorbidities, catheterization rates, and infection-control practices. Medium-sized regional hospitals—such as our centre—are rarely represented in international surveillance systems (e.g., EARS-Net, GLASS), resulting in a critical gap in data needed for empirical therapy and stewardship planning. Therefore, local microbiological surveillance remains a cornerstone of evidence-based treatment protocols. The aim of this study was to investigate the prevalence of specific causative agents of UTIs among hospitalized patients at the Doboj hospital over a three-year period (2021–2023). A secondary objective was to examine the resistance rate of *Klebsiella* spp., which showed the most notable increase as a UTIs pathogen during the observed period.

This study represents single-center surveillance data from a regional hospital and aims to provide actionable information for empirical treatment decisions, antimicrobial stewardship, and infection-control planning within similar healthcare settings.

## 2. Results

This retrospective study included 2183 urine culture samples of patients with UTIs, of which 61.3% were females, with a mean age of 72.27 ± 14.56 years. The youngest patient was 1 year old and the oldest was 94. There were 41 patients under 18 years of age (1.9%), while the most frequent age population was from 66 to 94 years of age (78%). A total of 657 urine samples (30.1%) were analyzed in 2021, 708 (32.4%) in 2022, and 818 (37.5%) in 2023. The overall incidence density of culture-confirmed UTIs was 29.4 per 1000 admissions, while the annual rates were 27.7, 28.4, and 31.9 per 1000 admissions in 2021, 2022, and 2023, respectively. Almost three-quarters of the urine samples were analyzed from patients hospitalized at the internal medicine department (74.3%), while 25.7% of urine samples were analyzed from patients hospitalized at the surgery department (Table 1).

Table 2 shows that the most commonly isolated bacterium was *E. coli* (29.2%), followed by *Klebsiella* spp. (24.2%) and *Enterococcus* spp. (19.8%). *E. coli* was significantly more frequently isolated from female patients than male patients (39.7% vs. 12.4%; *p* < 0.001) In contrast, *Acinetobacter* spp. (3.3% vs. 1%; *p* < 0.001), *Serratia* spp. (4.7% vs. 0.8%; *p* < 0.001), *Pseudomonas* spp. (10% vs. 6.5%; *p* = 0.005), *Enterococcus* spp. (24.5% vs. 13.6%; *p* < 0.001) and *Proteus* spp. (17.2% vs. 12.9%; *p* = 0.006) were all significantly more common in urine samples from male patients than from female patients.

*E. coli* was significantly more frequently isolated in patients younger than 18 years old when compared with older age groups (*p* < 0.001). In contrast, *Pseudomonas* spp. was significantly more commonly isolated in older patients than in younger groups (*p* = 0.041). Additionally, *Klebsiella* spp. showed the highest prevalence in the 19–45-year age group, with significantly increased isolation compared with both younger and older patients (*p* = 0.016) (Table 2).

*E. coli* was significantly (*p* < 0.001) more often isolated in 2022 (34.5%) and 2023 (31.5%) in comparison to 2021 (20.5%). *Klebsiella* spp. was significantly more often isolated in 2023 (28.9%) in comparison to previous years (2021—22.2%, and in 2022—20.8%, *p* < 0.001). The significant rise of isolation of *K. pneumoniae* was also seen in time, from 7.5% in 2021, to 7.3% in 2022 and 18.1% in 2023; *p* < 0.001). *Serratia* spp. was significantly (*p* < 0.001) more frequently isolated in 2021 (3.5%) and 2022 (3.5%) compared to 2023 (0.4%), while rates of *Pseudomonas* spp. and *Proteus* spp. significantly declined (*p* < 0.001 and *p* = 0.031) during observed period (from 2021 to 2023). We have also seen that *Enterococcus* spp. was more often isolated in internal medicine department in comparison to surgery department (21.2% vs. 15.9%; *p* = 0.006), while *K. pneumoniae* was isolated more often in surgery department in comparison to department of internal medicine (13.7% vs. 10.6%; *p* = 0.045) (Table 3).

Table 4 shows that there was no significant difference in the resistance of *Klebsiella* spp. to antibiotics between participants grouped by sex. However, it was observed that the resistance of *K. pneumoniae* to amoxicillin–clavulanic acid (ACA) (87.9% vs. 77.1%; *p* = 0.047), cefalexin (96.3% vs. 83.3%; *p* = 0.022), cefuroxime (97% vs. 87.2%; *p* = 0.030), ceftriaxone (89.1% vs. 71.6%; *p* = 0.016), and trimethoprim/sulfamethoxazole (TMP-SMX) (79.5% vs. 67%; *p* = 0.049) was significantly higher in male participants compared to female participants (Table 4).

Younger participants had a significantly higher prevalence of resistance of *Klebsiella* spp. to piperacillin–tazobactam (TZP), gentamicin, and amikacin compared to older participants group. Additionally, resistance of *K. pneumoniae* to ACA was significantly more common in patients between 19 and 65 years, while resistance of *Klebsiella* spp. and *K. pneumoniae* to ceftriaxone was significantly more common in the group of patients between 46 and 65 compared to younger and older patients (Table 5).

A significantly higher prevalence of resistance of *Klebsiella* spp. to cefotaxime was observed in urine samples from patients in the surgical department compared to those from the internal medicine department (Table 6).

A significant increase in resistance of *Klebsiella* spp. to TZP, cefalexin, cefuroxime, ceftriaxone, cefepime, imipenem, meropenem, gentamicin, amikacin, ciprofloxacin and TMP-SMX was noticed during observed time (all *p* values < 0.001) (Table 7). It is found that resistance of *Klebsiella* spp. to TZP, cefepime, imipenem, amikacin, gentamicin, and meropenem was low (1%, 7.7%, 0%, 1.1%, 8.8%, and 0.5%, respectively), but it significantly increased till 2023 (71.6%, 80.3%, 32.4%, 57.9%, 56.8%, and 59.59%, respectively). In contrast to *Klebsiella* spp., resistances of *K*. *pneumoniae* to cefalexin, cefuroxime, cefepime and ciprofloxacin were not significantly changed during observed period. However, resistances of *K. pneumoniae* to ACA, TZP, ceftriaxone, imipenem, meropenem, gentamicin, amikacin, and TMP-SMX had stable increase during observed time, with multiple magnification from 2021 to 2023 (51.6 to 100.0%, 66 to 85.2%, 25 to 79.1%, 4 to 39.5%, 16.4 to 56.7%, 35.2 to 63.9%, 30.8 to 60.2%, and 44.1 to 69.9%, respectively).

### Prevalence of Colistin-Resistant K. pneumoniae

At Doboj Hospital, the protocol of the microbiology laboratory does not include routine testing of *Klebsiella* isolates for colistin susceptibility. Testing is performed only on isolates that, based on the standard set of antibiotics, show indications of multidrug resistance or pan-resistance. During the observed period, an increase in the number of MDR *Klebsiella* isolates was recorded: only 1 (1%) in 2021, 20 (13.6%) in 2022, and 61 (35.5%) in 2023. Table 8 presents the prevalence of colistin-resistant *Klebsiella* isolates during the observed period.

## 3. Discussion

The most common causes of UTIs among patients in this study were *E. coli* (29.2%) and *Klebsiella* spp. (24.2%), followed by *Enterococcus* spp. (19.8%), *Proteus* spp. (14.6%), *K. pneumoniae* (11.4%), and *Pseudomonas* spp. (7.9%). Other species were isolated in fewer than 3% of urine samples. Over the observed period, shifts were noted in the frequency of certain pathogens, with a significant increase in the prevalence of *E. coli* and *Klebsiella* species, including *K. pneumoniae* and other members of the genus. The prevalence of all other uropathogens remained stable or declined. *E. coli* is globally recognized as the leading cause of UTIs, with its prevalence previously reported to exceed 70% [18,19]. The prevalence of *E. coli* as a causative agent in hospital-acquired urinary tract infections is lower than in community-acquired urinary tract infections and it is lower in seriously ill patients. Among patients with acute ischemic stroke, the reported proportions of UTIs attributable to *E. coli* are 26.8% in the United States, 45.5% in Turkey, 27.7% in Germany, and 41.9% in Thailand [20,21,22,23].

Additionally, we observed variations in pathogen prevalence across sex and age groups, suggesting that biological and demographic factors may influence the epidemiology of UTIs. For example, in our study *E. coli* was more frequently isolated in females and patients younger than 18 years. Similarly, another study found that *E. coli* was more prevalent in females, while its isolation rates were lower in males aged ≥60 years [24]. We also noted differences between departments. Enterococcus spp. were more frequently isolated in patients from the internal medicine department, whereas K. pneumoniae was more commonly detected in the surgical department. These differences may reflect variations in patient profiles and underlying conditions—for example, chronic comorbidities were more prevalent in internal medicine, while patients in the surgical department were typically in a postoperative state. Because urine sampling in the surgical department was performed after surgery, most patients had already been exposed to prophylactic antibiotic therapy. The observed increase in resistance may also reflect patterns of antibiotic use within the hospital, particularly the prophylactic use of β-lactams—primarily cefazolin—in surgical wards and broad-spectrum cephalosporins in internal medicine, both of which are recognized drivers of *Klebsiella* resistance. Departmental differences were also reported in another study, where the frequency of positive urine cultures and pathogen distribution varied significantly depending on the method of collection, hospitalization duration, and ward type, with catheter-based samples more often yielding nosocomial pathogens such as *Acinetobacter baumannii* and *Pseudomonas aeruginosa*, while midstream collections more frequently isolated community-associated organisms like *E. coli* and *Enterococcus* spp. [25].

In recent decades, global antibiotic consumption has increased significantly, resulting in changes and variations in the prevalence of UTIs pathogens across different communities and healthcare settings, along with a marked rise in bacterial resistance. While *E. coli* remains the leading cause of UTIs, the prevalence of other uropathogens, such as *Klebsiella*, *Enterococcus*, *Proteus*, and *Pseudomonas* species, has approached similar levels in certain populations, particularly in hospital-acquired infections [26]. Furthermore, the prevalence of UTIs caused by resistant strains, such as extended-spectrum beta-lactamase (ESBL) producing *E. coli* and *K. pneumoniae* [27] and extensively drug-resistant *K. pneumoniae* [28] is on the rise globally.

Our findings demonstrate concerning patterns of antimicrobial resistance among *Klebsiella* spp. and *K. pneumoniae*. While no significant differences in resistance were noted between sex for *Klebsiella* spp. overall, *K. pneumoniae* exhibited significantly higher resistance rates to several key antibiotics, including ACA, cefalexin, cefuroxime, ceftriaxone, and TMP-SMX, in male patients compared to females. Similarly with our findings, a decade-long analysis of carbapenem-resistant *K. pneumoniae* urinary isolates from hospitalized patients in South India reported a significantly higher overall isolation rate in males than females (59.5% vs. 40.5%), although certain years, such as 2015 and 2022, showed a higher proportion of carbapenem-resistant isolates in female patients, suggesting that sex-related differences in resistance may vary over time [29]. Age-related differences were also evident in our study, with younger participants showing significantly higher resistance of *Klebsiella* spp. to TZP, gentamicin, and amikacin, while *K. pneumoniae* resistance to ACA was more common in patients aged 19–65 years. Moreover, resistance of both *Klebsiella* spp. and *K. pneumoniae* to ceftriaxone was most pronounced in the 46–65-year age group. Previous studies have also found age-related variations in antibiotic resistance highlighting the need to tailor empirical therapy according to patient age [30]. Departmental variation was noted as well in our study, with cefotaxime resistance being significantly more prevalent among isolates from surgical patients compared to those from internal medicine. Similar findings have been reported elsewhere, where higher resistance rates to multiple antibiotics, including cefotaxime, were observed in surgical wards, likely reflecting the impact of prophylactic antibiotic use, greater illness severity, and prolonged hospital stays among surgical patients [31].

Over time, resistance of *Klebsiella* spp. and *K. pneumoniae* to a wide range of antibiotics, including some β-lactams, aminoglycosides, fluoroquinolones, and TMP-SMX, rose sharply in our study. Particularly alarming increases were observed for antibiotics such as TZP, cefepime, imipenem, meropenem, gentamicin, and amikacin. Notably, resistance rates that were initially low in 2021 escalated dramatically by 2023, underscoring the rapid progression of antimicrobial resistance within this setting. Similarly, a study involving *K. pneumoniae* urinary isolates from hospitalized patients in China reported a marked rise in resistance to carbapenems, TZP, cefepime, and amikacin [32]. The virulence of *K. pneumoniae*, driven by multiple factors such as capsule, lipopolysaccharide, siderophores, fimbriae, urease, and biofilm formation, promotes antimicrobial resistance and complicates treatment and persistence in the urinary tract [33]. Our findings are consistent with international antimicrobial resistance surveillance data. According to the most recent EARS-Net reports, *K. pneumoniae* ranks among the fastest increasing multidrug-resistant pathogens in Europe, with carbapenem resistance exceeding 30% in multiple countries, particularly in Southern and Eastern Europe [34,35]. WHO GLASS data similarly highlight rising resistance to third-generation cephalosporins, TZP, and carbapenems, with heterogeneity between regions but a consistent upward trajectory [35]. Studies from neighboring Balkan countries, including Serbia, Croatia, and North Macedonia, as well as from Romania and Greece, report comparable increases in MDR and XDR *K. pneumoniae* in hospital settings, especially among older adults and surgical or intensive-care patients [13,36,37]. Our results mirror these international trends and underscore the clinical urgency of strengthening stewardship, optimizing empirical therapy, and maintaining continuous surveillance in medium-sized hospitals such as ours.

The emergence of MDR *Klebsiella* isolates is especially worrisome in our setting, with prevalence increasing from only 1% in 2021 to over one-third of isolates by 2023. This trend was accompanied by a rise in colistin-resistant isolates, further narrowing available therapeutic options. These findings reflect not only the local challenges faced in managing UTIs but also align with global concerns regarding the rapid dissemination of MDR *K. pneumoniae*, emphasizing the urgent need for strengthened antimicrobial stewardship, infection control measures, and continuous resistance surveillance [38,39].

This study has several limitations. Its retrospective, single-center design may limit the generalizability of the findings and relies on the accuracy and completeness of existing records. Colistin susceptibility testing was performed only on isolates showing MDR or pan-resistance, potentially underestimating the true prevalence of colistin-resistant strains. Additionally, clinical outcomes such as treatment success or complications could not be assessed. Finally, molecular characterization of resistance mechanisms, such as ESBL or carbapenemase genotypes, could not be performed, limiting insights into the underlying genetic basis of observed resistance.

This single-center surveillance study is intended to support local antibiotic stewardship, update empirical treatment guidelines, and inform infection-control interventions. While molecular mechanisms of resistance were not investigated, this microbiology-based surveillance provides essential baseline data for clinical decision-making and future molecular studies.

## 4. Materials and Methods

### 4.1. Ethical Approval

This study was approved by the Ethics Committee of Saint Apostol Luka Hospital, Doboj, Republic of Srpska, Bosnia and Herzegovina (approval number 1013-1/24, issued on 9 February 2024).

### 4.2. Study Design and Setting

We conducted a retrospective cross-sectional study at Saint Apostol Luka Hospital, Doboj, Republic of Srpska, Bosnia and Herzegovina.

### 4.3. Data Source and Study Period

Data were obtained from the Department of Microbiology for the period from June to December 2024. Laboratory records from 2021, 2022, and 2023 were analyzed. Microbiological testing results were reviewed together with patient data, including age, sex, and the hospital department where the patient was admitted. All patient data were coded and kept fully confidential throughout the study period.

### 4.4. Participants and Eligibility Criteria

The study included all patients with clinical symptoms of UTIs and positive urine cultures showing ≥10^5^ colony-forming units (CFUs)/mL of a single uropathogen. Specimens with lower bacterial counts, mixed bacterial growth, or those collected incidentally during routine pre-operative or general check-ups, taken of patients without clinical symptoms of UTIs, were excluded. Patients with urinary catheters, pregnant women, and those who had used antibiotics within one month prior to sampling were also excluded.

For the final diagnosis of a UTI, midstream urine samples were collected in sterile containers. Inoculation was performed using sterile, calibrated disposable loops with a volume of 1 µL. The loop was dipped into the urine and withdrawn vertically; before inoculation, it was only checked whether urine was in the loop and not around it. The sample was then spread evenly over the entire surface of a urine culture medium using smooth strokes. The medium was incubated for 16–24 h at 35 ± 2 °C under aerobic conditions. The grown colonies were identified according to current microbiological procedures. The analysis took 24 h if the result was normal, and 48–72 h if identification and antimicrobial susceptibility testing were required.

### 4.5. Antibiotic Susceptibility Testing

Susceptibility testing of the isolated bacteria was performed according to EUCAST guidelines, using disc diffusion and automated instrumental methods, with broth microdilution applied specifically for colistin and vancomycin. Bacterial identification was performed using standard biochemical methods (VITEK and API) using the Vitek 2 Compact (BioMerieux, Salt Lake City, UT, USA).

### 4.6. Statistical Analysis

The methods of descriptive and analytical statistics were used for data description and analysis. Among the methods of descriptive statistics, measures of central tendency and measures of variability were used for numerical variables, namely: arithmetic mean with standard deviation. For the univariate analysis, differences in categorical variables were assessed using either the Chi-squared (χ^2^) test or Fisher’s exact test, depending on which test assumptions were met. The usual value of *p* < 0.05 was taken as the level of statistical significance of differences. All statistical analyses were performed using IBM SPSS Statistics Software version 24.0 for Windows (IBM Corp., Armonk, NY, USA).

## 5. Conclusions

This study highlights the evolving epidemiology and resistance patterns of uropathogens, with *E. coli* and *Klebsiella* spp. identified as the predominant causes of UTIs. Significant variations were observed across sex, age groups, and hospital departments, suggesting that both host-related and healthcare-associated factors contribute to pathogen distribution and resistance. The rapid increase in resistance among *Klebsiella* spp. and *K. pneumoniae* to multiple antibiotic classes was of particular concern. Given the limited therapeutic options, our results emphasize the urgent need for strengthened antimicrobial stewardship and ongoing surveillance of resistance trends. 

## Figures and Tables

**Table 1:** Time period of examination and distribution of patients with UTIs according to socio-demographic characteristics.

Variables	*n* (%)
Sex	
Male	844 (38.7)
Female	1339 (61.3)
Age (M ± SD), years	72.27 ± 14.56
1–18	41 (1.9)
19–45	32 (1.5)
46–65	408 (18.7)
66–94	1702 (78.0)
Department	
Surgery	561 (25.7)
Internal medicine	1622 (74.3)
Year	
2021	657 (30.1)
2022	708 (32.4)
2023	818 (37.5)
**Total**	2183 (100.0)

M—mean, SD—standard deviation.

**Table 2:** Distribution of isolated bacterial pathogens causing UTIs and differences in sex and age groups in the period between 2021 and 2023.

Isolated Bacteria	Sex *n* (%)		*p*	Age *n* (%)				*p*	Total *n* (%) 2183 (100)
	Male (*n* = 844)	Female (*n* = 1339)		1–18 (*n* = 41)	19–45 (*n* = 32)	46–65 (*n* = 408)	66–94 (*n* = 1702)		
*E. coli*	105 (12.4)	532 (39.7)	**<0.001**	33 (80.5)	11 (34.4)	100 (24.5)	493 (29.0)	**<0.001**	637 (29.2)
*Acinetobacter* spp.	28 (3.3)	13 (1.0)	**<0.001**	0(0.0)	0(0.0)	10 (2.5)	31 (1.8)	0.541	41 (1.9)
*Serratia* spp.	40 (4.7)	11 (0.8)	**<0.001**	0(0.0)	1 (3.1)	15 (3.7)	35 (2.1)	0.182	51 (2.3)
*Pseudomonas* spp.	84 (10.0)	89 (6.5)	**0.005**	0(0.0)	1 (3.1)	24 (5.9)	148 (8.7)	**0.041**	173 (7.9)
*Enterococcus* spp.	222 (26.3)	211 (15.4)	**<0.001**	2 (4.9)	4 (12.5)	81 (19.9)	346 (20.3)	0.068	433 (19.8)
*Proteus* spp.	145 (17.2)	173 (12.9)	**0.006**	1 (2.4)	3 (9.4)	61 (15.0)	253 (14.9)	0.127	318 (14.6)
*Klebsiella* spp.	219 (25.9)	310 (23.2)	0.138	5 (12.2)	11 (34.4)	117 (28.7)	396 (23.3)	**0.016**	529 (24.2)
*Klebsiella pneumoniae*	102 (12.1)	147 (11.0)	0.428	2 (4.9)	6 (18.8)	55 (13.5)	186 (10.9)	0.135	249 (11.4)

*p*—statistical significance was measured by χ^2^—chi square test or Fisher’s exact test, significant values are bolded.

**Table 3:** Distribution of isolated bacterial pathogens causing UTIs and differences in department and year groups.

Isolated Bacteria	Department *n* (%)		*p*	Year *n* (%)			*p*
	Surgery (*n* = 561)	Internal Medicine (*n* = 1622)		2021 (*n* = 657)	2022 (*n* = 708)	2023 (*n* = 818)	
*E. coli*	169 (30.1)	468 (28.9)	0.568	135 (20.5)	244 (34.5)	258 (31.5)	**<0.001**
*Acinetobacter* spp.	12 (2.1)	29 (1.8)	0.597	11 (1.7)	14 (2.0)	16 (2.0)	0.899
*Serratia* spp.	15 (2.7)	36 (2.2)	0.539	23 (3.5)	25 (3.5)	3 (0.4)	**<0.001**
*Pseudomonas* spp.	41 (7.3)	132 (8.1)	0.531	103 (15.7)	35 (4.9)	35 (4.3)	**<0.001**
*Enterococcus* spp.	89 (15.9)	344 (21.2)	**0.006**	126 (19.2)	137 (19.4)	170 (20.8)	0.689
*Proteus* spp.	93 (16.6)	225 (13.9)	0.117	112 (17.0)	106 (15.0)	100 (12.2)	**0.031**
*Klebsiella* spp.	141 (25.1)	388 (23.9)	0.563	146 (22.2)	147 (20.8)	236 (28.9)	**<0.001**
*Klebsiella pneumoniae*	77 (13.7)	172 (10.6)	**0.045**	49 (7.5)	52 (7.3)	148 (18.1)	**<0.001**

*p*—statistical significance was measured by χ^2^—chi square test or Fisher’s exact test, significant values are bolded.

**Table 4:** Difference in antibiotic resistance on uropathogens *Klebsiela* spp. and *Klebsiella pneumoniae* between sex categories in period from 2021 to 2023.

Antibiotics	*Klebsiella* spp. R				*p*	*Klebsiella pneumoniae* R				*p*
	Male		Female			Male		Female		
	*n*	%	*n*	%		*n*	%	*n*	%	
Ampicillin	168	98.8	211	99.1	0.820	91	100.0	109	100.0	1.000
Amoxicillin	90	97.8	123	98.4	0.756	20	100.0	28	100.0	1.000
ACA	143	84.1	168	79.2	0.224	80	87.9	84	77.1	**0.047**
TZP	110	71.9	124	70.9	0.836	74	84.1	74	83.1	0.865
Cefalexin	105	84.0	127	76.5	0.115	52	96.3	60	83.3	**0.022**
Cefuroxime	125	86.8	156	79.6	0.083	65	97.0	82	87.2	**0.030**
Ceftriaxone	97	72.9	119	68.0	0.349	49	89.1	53	71.6	**0.016**
Cefotaxime	32	72.7	68	68.7	0.627	12	92.3	32	84.2	0.464
Cefepime	109	71.2	132	73.3	0.671	71	82.6	76	80.9	0.767
Imipenem	49	33.1	69	39.7	0.224	35	42.2	43	48.9	0.380
Meropenem	79	52.7	87	49.7	0.596	49	57.6	52	58.4	0.917
Gentamicin	102	60.0	120	56.9	0.538	64	70.3	69	63.9	0.336
Amikacin	94	64.4	10	62.1	0.678	61	77.2	64	72.7	0.505
Ciprofloxacin	134	83.2	156	84.3	0.783	83	94.3	87	93.5	0.828
TMP-SMX	126	75.9	142	68.9	0.136	70	79.5	73	67.0	**0.049**

TZP—Piperacillin–tazobactam, ACA—Amoxicillin–Clavulanic acid, TMP-SMX—Trimethoprim/sulfamethoxazole, R—resistance, *p*—statistical significance was measured by χ^2^—chi square test or Fisher’s exact test, significant values are bolded.

**Table 5:** Difference in antibiotic resistance on uropathogens *Klebsiela* spp. and *Klebsiella pneumoniae* between age categories in period from 2021 to 2023.

Anti Biotics	*Klebsiella* spp. R								*p*	*Klebsiella pneumoniae* R								*p*
	1–18 Years		19–45 Years		46–65 Years		66–94 Years			1–18 Years		19–45 Years		46–65 Years		66–94 Years		
	*n*	%	*n*	%	*n*	%	*n*	%		*n*	%	*n*	%	*n*	%	*n*	%	
Amp	4	100	9	100	82	100	28	98.6	0.721	1	100	5	100	47	100	147	100	1.000
Amox	3	100	6	100	42	100	162	97.6	0.741	/	/	4	100	9	100	35	100	1.000
ACA	2	50.0	7	77.8	74	90.2	228	79.4	0.054	0	0.0	5	100	44	93.6	115	78.2	**0.010**
TZP	/	/	6	85.7	62	81.6	166	68.6	**0.005**	0	0.0	4	80.0	41	91.1	103	81.7	0.062
Cefalex	2	66.7	4	57.1	57	90.5	169	77.5	0.053	/	/	3	75.0	32	97.0	77	86.5	0.176
Cfu	2	66.7	6	66.7	65	91.5	208	80.9	0.086	/	/	4	80.0	36	97.3	107	89.9	0.251
Ceftriax	2	50.0	4	57.1	52	83.9	158	67.2	**0.049**	0	0.0	3	75.0	26	96.3	73	75.3	**0.023**
Cefotax	0	0.0	4	57.1	24	80.0	72	67.9	0.334	/	/	2	66.7	12	100	30	83.3	0.207
Cefep	1	50.0	6	85.7	64	82.1	170	69.1	0.107	/	/	4	80.0	43	91.5	100	78.1	0.128
Imi	/	/	3	42.9	29	38.7	86	36.3	0.573	0	0.0	2	40.0	22	50.0	54	44.6	0.731
Mero	/	/	3	42.9	44	57.9	119	49.8	0.180	0	0.0	2	40.0	29	64.4	70	56.9	0.411
Genta	3	75.0	6	66.7	58	70.7	155	54.2	**0.048**	1	100	5	100	35	74.5	92	63.0	0.159
Ami	/	/	6	85.7	53	71.6	145	60.4	**0.046**	/	/	4	80.0	34	79.1	87	73.1	0.716
Cipro	/	/	7	100	67	88.2	216	82.1	0.228	/	/	5	100	42	93.3	123	93.9	0.839
TMP-SMX	/	/	6	75.0	66	80.5	196	69.5	0.147	/	/	5	100	39	83.0	99	68.3	0.055

Amp—Ampicillin, Amox—Amoxicillin, TZP—Piperacillin–tazobactam, ACA—Amoxicillin–Clavulanic acid, Cefalex—Cefalexin, Cfu—Cefuroxime, Ceftriax—Ceftriaxone, Cefotax—Cefotaxime, Cefep—Cefepime, Imi—Imipenem, Mero—Meropenem, Genta—Gentamicin, Ami—Amikacin, Cipro—Ciprofloxacin TMP-SMX—Trimethoprim/sulfamethoxazole, R—resistance, *p*—statistical significance was measured by χ^2^—chi square test or Fisher’s exact test, significant values are bolded.

**Table 6:** Difference in antibiotic resistance to *Klebsiella* spp. and *Klebsiella pneumoniae* between department categories in period from 2021 to 2023.

Antibiotics	*Klebsiella* spp. R				*p*	*Klebsiella pneumoniae* R				*p*
	Surgery		Internal Medicine			Surgery		Internal Medicine		
	n	%	n	%		*n*	%	*n*	%	
Ampicillin	98	99.0	281	98.9	0.969	60	100.0	140	100.0	1.000
Amoxicillin	55	98.2	158	98.1	0.970	18	100.0	30	100.0	1.000
ACA	78	78.8	233	82.3	0.435	49	81.7	115	82.1	0.936
TZP	65	76.5	169	69.5	0.224	46	86.8	102	82.3	0.455
Cefalexin	66	81.5	166	79.0	0.643	37	86.0	75	90.4	0.465
Cefuroxime	73	83.0	208	82.5	0.930	43	87.8	104	92.9	0.290
Ceftriaxone	53	69.7	163	70.3	0.931	27	73.0	75	81.5	0.280
Cefotaxime	36	81.8	64	64.6	**0.039**	18	90.90	26	83.9	0.535
Cefepime	63	74.1	178	71.8	0.677	43	81.1	104	81.9	0.905
Imipenem	34	42.0	84	34.9	0.250	24	48.0	54	44.6	0.687
Meropenem	47	56.6	119	49.2	0.241	32	61.5	69	56.6	0.859
Gentamicin	64	64.6	158	56.0	0.135	41	68.3	92	66.2	0.768
Amikacin	57	69.5	147	61.0	0.167	39	79.6	86	72.9	0.363
Ciprofloxacin	79	87.8	211	82.4	0.235	51	92.7	119	94.4	0.657
TMP-SMX	72	75.8	196	70.8	0.346	45	77.6	98	70.5	0.310

TZP—Piperacillin–tazobactam, ACA—Amoxicillin–Clavulanic acid, TMP-SMX—Trimethoprim/sulfamethoxazole, R—resistance, *p*—statistical significance was measured by χ^2^—chi square test, significant values are bolded.

**Table 7:** Difference in antibiotic resistance to *Klebsiella* spp. and *Klebsiella pneumonia* in period from 2021 to 2023.

Antibiotics	*Klebsiella* spp. R						*p*	*Klebsiella pneumoniae* R						*p*
	2021		2022		2023			2021		2022		2023		
	*n*	%	*n*	%	*n*	%		*n*	%	*n*	%	*n*	%	
Ampicillin	90	83.3	92	93.9	87	98.9	**0.004**	47	100.0	52	100.0	148	100.0	1.000
Amoxicillin	87	82.9	83	93.3	82	98.8	**0.001**	5	100.0	19	100.0	29	100.0	1.000
ACA	86	78.2	79	72.5	68	77.3	0.682	32	51.6	46	79.3	118	100.0	**<0.001**
TZP	2	1.0	33	27.3	53	71.6	**<0.001**	31	66.0	39	66.1	109	85.2	**<0.001**
Cefalexin	51	57.3	56	47.5	64	82.1	**<0.001**	26	92.9	38	86.4	74	87.1	0.156
Cefuroxime	59	43.1	61	49.6	73	83.9	**<0.001**	40	87.0	48	92.3	99	89.2	0.541
Ceftriaxone	38	24.1	46	33.8	68	77.3	**<0.001**	6	25.0	30	65.2	72	79.1	**<0.001**
Cefotaxime	57	41.0	56	43.8	/	/	0.883	37	78.7	44	75.9	/	/	0.779
Cefepime	14	7.7	33	27.3	61	80.3	**<0.001**	33	80.5	42	84.0	105	78.4	0.275
Imipenem	0	0.0	13	9.2	24	32.4	**<0.001**	3	4.0	23	32.4	49	39.5	**<0.001**
Meropenem	1	0.5	18	13.2	44	59.5	**<0.001**	11	16.4	23	32.4	72	56.7	**<0.001**
Gentamicin	16	8.8	39	26.2	50	56.8	**<0.001**	25	35.2	39	60.0	94	63.9	**<0.001**
Amikacin	2	1.1	29	22.1	44	57.9	**<0.001**	16	30.8	32	57.1	74	60.2	**0.003**
Ciprofloxacin	41	27.2	51	44.3	69	84.1	**<0.001**	39	95.1	47	92.2	123	93.2	0.762
TMP-SMX	48	32.9	57	45.6	68	81.0	**<0.001**	30	44.1	41	67.2	102	69.9	**<0.001**

TZP—Piperacillin–tazobactam, ACA—Amoxicillin–Clavulanic acid, TMP-SMX—Trimethoprim/sulfamethoxazole, R—resistance, *p*—statistical significance was measured by χ^2^—chi square test or Fisher’s exact test, significant values are bolded.

**Table 8:** Prevalence of colistin-resistant *Klebsiella* isolates during the observed period.

Antibiotics	*Klebsiella* spp. R						*p*	*Klebsiella pneumoniae* R						*p*
	2021		2022		2023			2021		2022		2023		
	n	%	n	%	*n*	%		*n*	%	*n*	%	*n*	%	
Colistin	0	0	9	6.1	24	13.9	**<0.001**	0	0	6	11.5	19	17.1	**<0.001**

R—resistance, Fisher’s exact test, significant values are bolded.

## Data Availability

All data supporting the findings can be found within the manuscript.

## Abbreviations

The following abbreviations are used in this manuscript:

UTI	Urinary tract infection
MDR	Multidrug resistance
PDR	Pandrug resistance
ACA	Amoxicillin–clavulanic acid
TMP-SMX	Trimethoprim/sulfamethoxazole
TZP	Piperacillin–tazobactam
R	Resistance
CFU	Colony-forming units
